# Implications of Oxidative Stress and Potential Role of Mitochondrial Dysfunction in COVID-19: Therapeutic Effects of Vitamin D

**DOI:** 10.3390/antiox9090897

**Published:** 2020-09-21

**Authors:** Natalia de las Heras, Virna Margarita Martín Giménez, León Ferder, Walter Manucha, Vicente Lahera

**Affiliations:** 1 Departamento de Fisiología, Facultad de Medicina, Universidad Complutense, 28040 Madrid, Spain; vlahera@ucm.es; 2 Instituto de Investigaciones en Ciencias Químicas, Facultad de Ciencias Químicas y Tecnológicas, Universidad Católica de Cuyo, San Juan 5400, Argentina; vmartin@uccuyo.edu.ar; 3 Universidad Maimónides, Buenos Aires 1405, Argentina; ferder.leon@maimonides.edu; 4 Área de Farmacología, Departamento de Patología, Facultad de Ciencias Médicas, Universidad Nacional de Cuyo, Mendoza 5500, Argentina; wmanucha@fcm.uncu.edu.ar; 5 Instituto de Medicina y Biología Experimental de Cuyo (IMBECU), Consejo Nacional de Investigaciones Científicas y Tecnológicas (CONICET), Mendoza 5500, Argentina

**Keywords:** SARS-CoV-2 infection, oxidative stress, mitochondrial dynamics, vitamin D, renin-angiotensin-aldosterone system, COVID-19, inflammation, cytokines

## Abstract

Due to its high degree of contagiousness and like almost no other virus, SARS-CoV-2 has put the health of the world population on alert. COVID-19 can provoke an acute inflammatory process and uncontrolled oxidative stress, which predisposes one to respiratory syndrome, and in the worst case, death. Recent evidence suggests the mechanistic role of mitochondria and vitamin D in the development of COVID-19. Indeed, mitochondrial dynamics contribute to the maintenance of cellular homeostasis, and its uncoupling involves pathological situations. SARS-CoV-2 infection is associated with altered mitochondrial dynamics with consequent oxidative stress, pro-inflammatory state, cytokine production, and cell death. Furthermore, vitamin D deficiency seems to be associated with increased COVID-19 risk. In contrast, vitamin D can normalize mitochondrial dynamics, which would improve oxidative stress, pro-inflammatory state, and cytokine production. Furthermore, vitamin D reduces renin–angiotensin–aldosterone system activation and, consequently, decreases ROS generation and improves the prognosis of SARS-CoV-2 infection. Thus, the purpose of this review is to deepen the knowledge about the role of mitochondria and vitamin D directly involved in the regulation of oxidative stress and the inflammatory state in SARS-CoV-2 infection. As future prospects, evidence suggests enhancing the vitamin D levels of the world population, especially of those individuals with additional risk factors that predispose to the lethal consequences of SARS-CoV-2 infection.

## 1. Introduction

In terms of public health, the COVID-19 pandemic is the biggest challenge that humanity has had to face in the last decade. Many other viruses have put the health of the population around the world at risk, but few with the high degree of contagiousness of SARS-CoV-2 [1]. Despite the efforts of multiple scientific groups from different nationalities, and the promising results obtained so far, the possibility of having a vaccine against SARS-CoV-2 infection seems to be quite far yet [2].

In this sense, it is essential to deepen the knowledge about the therapeutic targets and the properties of active compounds already known that can help to prevent, combat, or alleviate the symptoms of severe acute respiratory syndrome (SARS) caused by COVID-19 pathology [3,4].

The exacerbated and uncontrolled oxidative stress is one of the main causes of morbidity and mortality by this respiratory syndrome in patients susceptible to this viral infection. For this reason, the analysis of the role of cellular organelles and substances that are directly involved in the regulation of oxidative stress is essential [5,6]. It is well-described that the antioxidant effects of vitamin D may be very useful in the prevention and attenuation of COVID-19 symptoms, and that a deficient vitamin D status would confer susceptibility to morbidity and mortality by SARS-CoV-2 [7,8,9].

In this review, the role of mitochondria and vitamin D is extensively examined in the context of SARS-CoV-2 infection, with the aim of proposing a potential therapeutic alternative for this pandemic that has become a nightmare for the world population, while waiting for the development of a vaccine.

## 2. Mitochondrial Dynamics: Role in Cell Physiology

**Figure 1:**
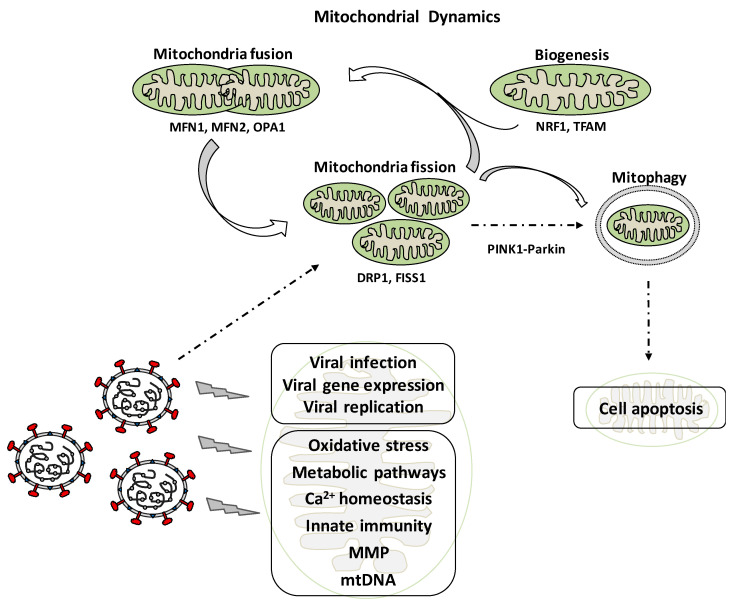
Effects of viral infection on mitochondrial dynamics, the viral life cycle, and various aspects associated with the internal metabolism of the mitochondria as well as its physiological processes.

Mitochondria are cell organelles that play a key role in the maintenance of cellular homeostasis. They are involved in numerous functions and signaling pathways such as energy metabolism, innate immunity, calcium homeostasis, apoptosis, aging, and others [10]. Mitochondria are not static, but dynamic organelles involved in adaptation to changes in the metabolic environment of cells as well as their survival or death, in order to maintain the quality of mitochondrial function. Mitochondrial dynamics includes fusion, fission, and mitophagy processes as well as biogenesis (Figure 1). These processes are not only involved in physiological situations, but also in pathological ones such as cancer, cardiovascular, neurodegenerative, and metabolic diseases [11].

The fusion process is the union of two mitochondria in a single one [12]. Mitochondrial fusion maintains an intact mitochondrial DNA (mtDNA) and participates in matrix metabolites exchanging. The fusion process is mediated by Mitofusin 1 (MFN1) and MFN2 (outer-membrane proteins), and optic atrophy-1 (OPA1), three large GTPases of the dynamin superfamily that mediates inner-membrane fusion [13]. Mitochondrial fission is mediated by Dynamin-related Protein 1 (DRP1), which is regulated by phosphorylation of DRP1 and acetylation and S-nitrosylation. The fission process is the division of mitochondria into smaller ones, and helps in sorting damaged or impaired mitochondria that are further eliminated by a mitophagy [14]. Mitophagy is a specialized form of autophagy in order to degrade abnormal mitochondria, and is related to cell apoptosis. It is also a critical process for maintaining proper cellular functions, and involves the formation of an autophagosome that fuses with lysosomes to degrade mitochondria [15]. Mitophagy is mediated either by the PINK1-Parkin signaling pathway or the mitophagic receptors BCL2 Interacting Protein 3 (BNIP3) and BNIP3-like, also known as NIX [16,17]. Changes in mitochondrial membrane potential and other stress situations result in the accumulation of PINK1 on the outer membrane, which phosphorylates numerous proteins including PARKIN and E3 ubiquitin ligase. Activated PARKIN results in activation of the ubiquitin-proteasome system that allows for the degradation of a number of membrane proteins external (MFN 1 and 2), thus enhancing mitochondrial fragmentation (Figure 1). The actual level of mitochondria in cells depends on the balance between biogenesis, and the degradation. Mitochondrial biogenesis is stimulated by the peroxisome proliferator-activated receptor γ co-activator 1 α (PGC-1α), which modulates the expression of nuclear respiratory factor 1 (NRF1). NRF1 stimulates the expression of mitochondrial transcription factor A (TFAM), which is a final effector activating the duplication of mitochondrial DNA molecules [16].

## 3. Coronaviruses and Acute Respiratory Syndromes 

Coronaviruses (CoV) are a broad family of RNA viruses that can cause respiratory, gastrointestinal, hepatic, and neurological diseases in animals, but only few known coronaviruses frequently cause disease in humans. The infections start in animals and spread to people. Four coronaviruses produce symptoms similar to those of the common cold, but three can cause severe and even fatal respiratory infections in humans: SARS-CoV-2, MERS-CoV, and SARS-CoV [18]. SARS-CoV-2 is a new coronavirus identified as the cause of the 2019 coronavirus disease (COVID-19) that started in Wuhan, China at the end of 2019, and has spread worldwide. MERS-CoV was identified in 2012 as the cause of Middle East respiratory syndrome (MERS). SARS-CoV was identified in 2002 as the cause of an outbreak of severe acute respiratory syndrome (SARS) that started in China in late 2002. SARS-CoV-2 exhibits significant person-to-person transmission through contact with secretions from respiratory droplets or through contact with contaminated surfaces [19].

Some people with COVID-19 disease may have few or no symptoms, although others become seriously ill and die. Symptoms may include fever, cough, dyspnea, chills or tremors, tiredness, muscle pain, headache, odynophagia, loss of smell and/or taste, nausea, vomiting, and diarrhea. The incubation time is between two and 14 days after exposure to the virus [20]. The risk of severe disease and death during SARS-CoV-2 infection increases with age and in people with several pathologies like cardiac, respiratory renal, hepatic diseases, diabetes, or obesity. Severe COVID-19 is characterized by dyspnea, hypoxia, and extensive lung impairment, which can lead to respiratory failure shock and death. Frequent complications of COVID-19 are arrhythmias, cardiomyopathy, thromboembolism and pulmonary embolism, disseminated intravascular coagulation, hemorrhage and arterial clot formation, sepsis, shock, and multi-organ failure [21].

## 4. Mitochondrial Dynamics and Viral Infection

The participation of mitochondria in innate immune signaling and their implication in viral infections is an emerging field of investigation. It appears that mitochondria are targeted by viral proteins and also influenced by alterations of the physiological cellular environment. It has been shown that during viral pathogenesis deregulation of calcium homeostasis, endoplasmic reticulum stress, oxidative stress, and hypoxia exist, and viruses alter mitochondrial dynamics for the progression of infection (Figure 1) [15,22]. The understanding of virus/host interactions will be useful for the knowledge of viral infection pathogenesis and to design new antiviral therapeutic strategies, mainly after the COVID-19 pandemic.

The steps of the viral life cycle include entry, translation, replication, assembly, and egress [23]. This dynamic process induces host cellular reorganization, which includes localization of the viral proteins to an appropriate subcellular compartment. This is a viral strategy to modify the host machinery and pathways to establish and perpetuate an infection [22,24]. Thus, viruses develop strategies to survive and proliferate in cells by targeting specific cell organelles (nucleus, mitochondria, endoplasmic reticulum, peroxisomes, and lipid droplets), which play an important role in innate immunity and host defense [15].

The viral infection causes alterations that can affect mitochondrial dynamics at different levels. As previously mentioned, mtDNA is crucial for synthesizing enzymes involved in respiratory chain and optimal functioning of the organelle. Thus, viruses damage mtDNA to evade immune defense of the host cell [25]. It has been shown that lymphoma cells infected with hepatitis C virus (HCV) suffered mtDNA depletion [26]. Expression of UL12.5, an amino-terminally truncated UL12 isoform of HSV-1 has been shown to induce mtDNA degradation [27].

Mitochondrial membrane potential (MMP) provides energy for ATP synthesis and is essential for adequate functioning of mitochondria. Increased MMP induces apoptosis, while decreased MMP prevents apoptosis [25]. Mitochondrial permeability transition pore (MPTP) is responsible for the maintenance of MMP and is vital for mitochondrial homeostasis. Alterations of MPTP leads to osmotic water flux, swelling, outer membrane rupture, and release of proapoptotic factors leading to cell death [28]. Viral infections result in an altered MPTP [29]. In general, viruses decrease MMP to prevent cell death in order to promote their replication. However, in advanced levels of infection, they may trigger an increase in MMP to release the progeny virions by apoptosis [22]. It has been reported that M11L protein of myxoma poxvirus prevents the loss of MMP in kidney cells, HeLa cells, THP-1 human monocytes, and T lymphocytes [30]. In contrast, R protein of HIV-1 induces the loss of MMP in lymphoblasts and T lymphocytes, resulting in apoptosis [31].

Viruses alter most of the mitochondrial metabolic pathways to maintain cellular energy homeostasis in order to ensure efficient replication and to avoid mitochondrial antiviral response [32]. Some viruses increase aerobic glycolysis and use glucose as an energy source, in order to maintain the availability of fatty acids, lipids, and nucleotides for their replication [33,34]. In fact, the leukemia virus infection on fibroblasts resulted in a significant increase in glucose uptake and lactic acid production [35]. Several types of cells upon viral infection enhances the glycolytic pathway and directs the supply of carbon from glucose to the stricarboxylic acid cycle, which facilitates fatty acid synthesis. However, it has also been shown that viral infection directs the central carbon metabolism to produce pyrimidine nucleotide components [36].

As mentioned before, mitochondria are involved in cell calcium homeostasis, which is of vital importance for several functions. Many viruses also alter mitochondrial calcium homeostasis to attain their needs during its life cycle [25]. It has been reported that human cytomegalovirus (HCMV) infection causes calcium influx into mitochondria from endoplasmic reticulum [37]. In addition, expression of 2B protein of coxsackievirus reduced signaling of Ca2þ between the endoplasmic reticulum and mitochondria, resulting in the suppression of apoptosis [38].

Viruses modify the intracellular number and distribution of mitochondria either to get energy for replication or to prevent the release of apoptosis mediators [10]. It has been reported that hepatitis B virus (HBV) infection produces mitochondrial fission and subsequent mitophagy. This effect appears to be due to the induction of mitophagosome formation, which leads to mitophagy and apoptosis prevention, thus facilitating persistent infection [39]. In lung cancer cells, Newcastle disease virus (NDV) induces mitophagy, which promotes replication by preventing caspase dependent apoptosis [40]. Degradation of the mitochondrial antiviral-signaling protein (MAVS) is another way to enhance mitophagy in order to attenuate the antiviral immune response, as shown in measles virus infection in lung cancer cells [41]. Moreover, induction of mitophagy resulting in suppression of type 1 interferon (IFN) response was reported in HEK293T infected by human parainfluenza virus type 3 (HPIV3) [42].

The mechanisms by which viruses evade the innate immunity system is poorly understood [43]. A recent study revealed that SARS-CoV protein open reading frame-9b (ORF-9b) promotes degradation of Drp1, leading to mitochondrial fusion [44]. This reduction was sensitive to proteasome inhibition, but unaffected by inhibition of autophagy. Lowered Drp1 expression was also associated with impaired MAVS signaling. This is contrast to the notion that MAVS signaling and IFN production are enhanced and dampened by mitochondrial fusion and fission, respectively [45].

## 5. Oxidative Stress and Interaction with the Immune System in the Worsening of Viral Infection Symptoms

The term oxidative stress refers to a disturbance in the oxidant–antioxidant balance, leading to potential cellular damage. This imbalance could result from a lack of antioxidant capacity or an overabundance of reactive oxygen species (ROS). In general, cellular metabolism produces ROS as a by-product of the normal aerobic metabolism by a variety of enzymes in mitochondria, endoplasmic reticulum, and peroxisome compartments, and simultaneously, the oxide is removed to keep the balance [46]. ROS are reactive chemical species containing oxygen, and the main ROS are the superoxide anion (O_2_^•−^) and its derivatives hydrogen peroxide (H_2_O_2_) and hydroxyl radical (•OH), which have been integrated in an essential way in intra- and intercellular signaling. To cite some examples, ROS are mediators of essential cellular functions such as gene expression, protein phosphorylation, activation of transcription factors, DNA synthesis, or cell proliferation. Ultimately, the biological impact of these molecules will be determined by the amount of ROS, cellular defenses, and cellular adaptive capacity. However, ROS play a bivalent role because if their production is uncontrolled, the result is an indiscriminate oxidative attack on lipids, proteins, and cellular DNA favoring tissue damage, inflammation response, and cell death [46,47,48].

ROS are produced during viral infections and significantly affect both the production of oxidizing agents and the synthesis of antioxidant enzymes. The reproduction or replication of some pathogens is enhanced in the oxidative environment and Peterhans [49] published the first evidence that a virus could induce oxidative stress by increasing the ROS levels. Later, other studies showed that many DNA and RNA viruses and retroviruses could cause cell death by generating ROS [50,51,52]. These are generated during the process of immunological activity by myeloperoxidase (MPO), NADPH oxidase (NOX), and inducible nitric oxide synthase (iNOS) to fight pathogens. As a consequence, gene expression, cell adhesion, metabolism, cell phases, and the various possibilities of cell death are modulated. Therefore, cell and tissue damage due to both the viral infection and the ROS produced would contribute to the pathophysiology [53].

Mitochondria are the major source of ROS in cells. Balance between ROS production and scavenging is essential for the optimal functioning of cells [54]. Viral infections affect the production of mitochondrial ROS because viruses can induce or inhibit various mitochondrial processes in a highly specific way so that they can replicate and produce progeny [25]. In general, viruses increase the production of ROS, which activates certain host cellular pathways that favor viral replication. During the process of interaction with the host, they can be produced by biotransformer enzymes such as cytochrome P450, spermine oxidase, and xanthine oxidase. However, it seems that both increasing and decreasing oxidative stress could be used as a survival strategy by viruses [55]. Furthermore, in some viral infections, the inhibition of the expression of primary antioxidant enzymes such as superoxide dismutase (SOD), glutathione peroxidase, and catalase as well as non-enzymatic antioxidants such as vitamin C, carotenoids, minerals, and cofactors occurs as a consequence of the action of viral regulatory proteins on cellular activity [52,56,57]. Alterations in the body’s antioxidant defense system have been observed in various tissues of patients infected with a retrovirus such as the human immunodeficiency virus (HIV) in relation to SOD, ascorbic acid, glutathione, and selenium, among others [52,58]. In infected mice, respiratory syncytial virus (RSV) decreased SOD and glutathione activity, in addition to increasing MPO activity, nitric oxide (NO) production from iNOS, and •OH levels [59]. Similarly, the activation of nuclear factor E2-related factor 2 (Nrf2) is considered an effective antioxidant defense mechanism used by host cells to counteract oxidative stress [60]. Nrf2 has been identified as the master regulator of several hundred genes involved in the antioxidant defense response to virus, bacteria, and parasite infections. However, not much is known about the role of Nrf2 in infection by certain viruses such as respiratory viral infections. Previous studies have shown that heme oxygenase (HO)-1, one of the phase II detoxifying enzymes inducible through the activation of Nrf2, participates in an important way in the cytoprotective function against inflammation and oxidative stress during viral infection [60,61].

During an infection, some cells of the immune system including macrophages, neutrophils, and dendritic cells are activated by generating enzymes such as MPO to produce ROS as hypochlorous acid with potential virucidal and bactericidal activity that are generated to annihilate the pathogens contained in phagosomes, but they, and some derived metabolites, also constitute signals that coordinate the actions of various cell types. NOX has a similar function but produces superoxide anion. The iNOS that generates NO is another of the ROS generating enzymes where during infections, it is abundant in macrophages and other leukocytes. NO is not very toxic, but when it reacts spontaneously with superoxide, peroxynitrite is produced, which is almost 1000 times more toxic than the radical •OH. Peroxynitrite is very effective in the annihilation of pathogens although it is highly toxic [62,63]. Oxidative stress can also be initiated by metabolic activity of the pathogen or during alteration of host metabolism as a consequence of interaction with the pathogen. In this sense, numerous molecular components of pathogenic organisms in the biological medium must be biotransformed for their elimination by enzymes such as some isoforms of cytochrome P450 (CYP3A4) that in their majority generate O_2_^•−^ [64]. Other intracellular enzymes such as spermine oxidase also interact with biomolecules of pathogens and generate ROS [65]. Some biomolecules of pathogens interact with the endoplasmic reticulum, and in the mitochondria enhance their activity and alter the membrane potential, thereby modulating the generation of ROS [10]. Oxidative stress can induce not only molecular damage, but also disruption of regulatory processes as these oxidized products can interact with molecular damage pattern recognition receptors and modulate transcription factor activation and expression of genes. As a consequence, cell death processes such as necrosis, apoptosis, or pyroptosis, which has characteristics of the previous two, can occur or otherwise cause survival mechanisms [52,66,67]. Thus, ROS can facilitate or even promote viral replication, depending on the type of cells and the type of virus involved.

In addition to ROS release due to virus-induced phagocyte activation, these activated cells of the immune system can release pro-oxidant cytokines such as tumor necrosis factor (TNF) [68]. Generally, the oxidative environment can enhance the activation of redox-sensitive transcription factors such as nuclear transcription factor kappa B (NF-κB) and hypoxia-inducible factor 1 alpha (HIF-1α) [68,69]. In this sense, TNF stimulates the release of NF-κB from cytoplasmic inhibitory protein IkB. As a result, this transcription factor translocates to the nucleus and binds to DNA by inducing transcription of cellular and/or viral genes and therefore increased viral replication [70]. Furthermore, viral infections develop local hypoxia where phagocytic cells would activate HIF-1α, and this factor would stimulate the expression of important genes related to phagocyte function. Several studies have shown that NF-κB is one of the main links between innate immunity and the hypoxic response through its transcriptional control of HIF-1α expression [71].

In general, SARS-CoV-2 infection is associated with oxidative stress, the proinflammatory state, cytokine production, and cell death [5,72,73]. In experimental animal models of SARS, an increase in ROS levels and an alteration of antioxidant defense were demonstrated during SARS-CoV infection [74]. In SARS-CoV 3CLpro (a viral protease) cell models, it significantly increased ROS production and caused a pro-inflammatory and apoptotic state [75]. In this sense, innate immunity would in turn participate by activating various transcription factors such as NF-κB, which would cause an exacerbated pro-inflammatory response of the host [5,72,73].

However, one of the most important effects is that it activates the innate immune system, generating an excessive response that could be related to greater lung injury and worse clinical evolution. Recent studies have described the activation circuit of this immune pathway from the activation of CD4+ and CD8 + T helper (Th) lymphocytes in patients with SARS-CoV-2 pneumonia. Furthermore, they describe a positive correlation between the proportion of IL-6-producing CD4 + T cells and granulocyte-macrophage colony-stimulating factor (GM-CSF) and the severity of cases of COVID-19 [76]. Other studies have observed the presence of elevated levels of IL-6 and other proinflammatory cytokines in patients with severe COVID-19 [76,77,78]. This hyperactivation, however, is insufficient to control the infection and leads to a lymphocyte depletion associated with increased tissue damage. This hyperactivation has been called cytokine release syndrome (CRS), which would be associated with SARS or acute respiratory distress syndrome, which has been described as the main cause of mortality from COVID-19. CRS occurs when large numbers of leukocytes (neutrophils, macrophages, and mast cells) are activated and release a high concentration of proinflammatory cytokines [79]. CRS was initially described as an adverse effect of monoclonal antibody therapies, and is also common in chimeric antigen receptor (CART) T-cell therapies [80]. The main cytokines involved in CRS pathogenesis include interleukin 6 (IL-6), IL-10, IFN, monocyte chemotactic protein 1 (MCP-1), and GM-CSF; other cytokines such as TNF, IL-1, IL-2, IL-2 receptor, and IL-8 have also been described during CRS. This hyperactivation state has also been observed in other viral infections such as SARS, MERS, or Ebola, in which the altered pathways are different.

## 6. Effects of Vitamin D in the Attenuation of Mitochondrial Oxidative Stress

Although vitamin E is one of the most famous and well-investigated radical-scavenging antioxidants, vitamin D may also function as a powerful antioxidant, even showing in many circumstances a higher effectiveness than that observed with vitamin E supplementation [81]. Vitamin D may act as an antioxidant by mitochondrial function stabilization. For example, it is known that cyanide causes neurotoxicity and neuronal cell death through mitochondrial dysfunction, which at low doses of cyanide is potentiated by the induced upregulation of uncoupling protein-2 (UCP-2). In rat primary cortical cell culture, vitamin D was able to attenuate the mitochondrial dysfunction provoked by cyanide. This effect was reflected through the restoration of mitochondrial membrane potential and cellular ATP by the downregulation of UCP-2 through the inhibition of NF-kB and the reduction in oxidative stress [82].

One study also demonstrated that calcitriol, the active form of vitamin D, reversed the oxidative cardiac injury induced by isoproterenol in a rat model. These effects were mediated by the reduction in H_2_O_2_ levels accumulated in cardiac tissue and the increase in superoxide dismutase and catalase activities as antioxidant mechanisms [83]. Paricalcitol, an analogous of vitamin D, which acts as a ligand of vitamin D receptors (VDR), caused renal protection in spontaneously hypertensive rats through the prevention of mitochondrial injury and a reduction in NOX activity (pro-oxidative enzyme). These protective effects were mediated by an increase in the intracellular levels of heat shock protein 70 (an antioxidant chaperone at intracellular but not extracellular level) and a decrease in angiotensin II (Ang II) type 1 receptors in renal cortex cells from these animals [84]. Another beneficial effect of vitamin D on cardiovascular health has also been reported, specifically preventing the death of endothelial cells. Vitamin D may exert this action through a decrease in cellular apoptosis and autophagy-mediated by the adequate maintenance of mitochondrial function and the reduction in O_2_^•−^ production, among other protective mechanisms [85]. Monoamine oxidase is a mitochondrial enzyme that generates H_2_O_2_ as a degradation product from its corresponding substrates, causing a pro-oxidative state. This enzyme is found induced in the aortas of diabetic rats, and vitamin D demonstrated to modulate its expression, causing an improvement of endothelial dysfunction induced by diabetes. Vitamin D considerably restored vascular function, attenuated oxidative stress, and reduced monoamine oxidase expression in vascular preparations from diabetic rats [86].

As a result of a clinical trial performed with pregnant women, it has also been suggested that vitamin D could act as a competitive inhibitor of cytochrome P450scc in placental cells, avoiding the lipid peroxidation and oxidative stress phenomenon that usually contributes to the development and pathogenesis of preeclampsia. In this sense, it was observed that women suffering from this condition had low vitamin D plasma levels, whereas vitamin D supplementation was able to prevent the onset of preeclampsia or treat it [87]. Moreover, in an animal model of pregnant rats with preeclampsia, vitamin D was also able to reduce the placental level’s oxidative stress. This effect decreased fetal mortality and altered parameters observed in these female rats such as mean blood pressure and urine micro albumin, among others [88].

Calcitriol may also prevent multiple alterations at the brain level associated with hyperhomocysteinemia. It is known that high plasma levels of homocysteine could condition the development of different neurodegenerative disorders. In vitro studies on cerebral cortices from rats pre-treated with calcitriol and exposed to a mild concentration of homocysteine, demonstrated that altered bioenergetics parameters and impaired mitochondrial functions promoted by homocysteine were significantly attenuated by pre-treatment with calcitriol. Specifically, calcitriol reduced the concentration of ROS and lipid peroxidation and increased the antioxidant enzyme activity, preventing changes in mitochondrial brain cell [89]. This same protective antioxidant effect of vitamin D against hyperhomocysteinemia was observed in heart tissue, where the accumulation of homocysteine may contribute to the development of cardiovascular disease [90]. Vitamin D, combined with lipoic acid, reduced the mitochondrial dysfunction in primary mouse astrocytes with oxidative stress induced by H_2_O_2_. This action confirms that vitamin D could also act as a drug or an adjuvant in the prevention or delay of aging and its related pathologies [91]. The antioxidant potential of vitamin D was also observed in Alzheimer’s disease induced by streptozotocin in animals. Vitamin D reduced neuronal oxidative stress as well as mitochondrial aberrations provoked in these animals. Therefore, vitamin D could also be useful in the prevention or treatment of Alzheimer’s disease and other neurobehavioral disorders associated with oxidative stress [92].

Calcipotriol, a synthetic analog of calcitriol, demonstrated its antioxidant potential on melanocytes subjected to oxidative damage. This finding is of particular interest because melanocyte loss by oxidative stress usually triggers the development of vitiligo. In this study, oxidative stress was induced in human melanocytes through the treatment with H_2_O_2_. Calcipotriol was able to reduce the concentrations of malondialdehyde, increase the levels of SOD, and suppress the reduction of the MMP. Likewise, calcipotriol reduced the ultrastructural mitochondrial damage in melanocytes under oxidative stress [93].

During peritoneal fibrosis, where the oxidative stress has a crucial role, an epithelial-to-mesenchymal transition of mesothelial cells is produced from peritoneum mediated by TGF-β1. In this sense, paricalcitol reduced the epithelial-to-mesenchymal transition by TGF-β1 through the modulation of oxidative stress associated with mitochondrial production of NOX [94]. In an experimental model of cardiac dysfunction induced by chronic immobilization stress, vitamin D treatment provoked an increase in tissue reserves of glutathione, ATP, SOD, and cardiolipin, which were initially decreased. Moreover, vitamin D also caused a decrease in malondialdehyde concentrations, which was elevated due to mitochondrial dysfunction and oxidative stress associated with chronic stress [95].

The administration of vitamin D, both separately and in combination with polyunsaturated fatty acids, caused attenuation of exacerbated oxidative stress and lipid peroxidation processes carried out in the liver from rats with cancer. On the other hand, these treatments restored the hepatic mitochondrial functional capacity in these animals, reducing the O_2_^•−^ generation and mitochondrial cytochrome c release [96]. Continuing with liver diseases, it is known that oxidative stress produced during alcohol metabolism plays a vital role in the development of the alcoholic liver disease. In this sense, it has been proven that vitamin D induces the expression of nuclear factor erythroid 2-related factor 2, which could transcriptionally upregulate the expression of aldehyde dehydrogenase 2 in order to collaborate in alcohol metabolization, exerting an antioxidant effect [97].

Annulus fibrosus cells isolated from young rat lumbar discs and subjected to different concentrations of H_2_O_2_ were pre-treated with vitamin D to evaluate its antioxidant potential for the prevention of intervertebral disc degeneration. In this regard, vitamin D caused an increase in both cell viability and MMP and its ATP content. Furthermore, this pre-treatment provoked a reduction in ROS production and preserved the enzymatic activity at the oxidative respiratory chain level, exerting mitochondrial protection against the damage induced by H_2_O_2_. Furthermore, the ablation of VDR in these cells avoided the mentioned protective effects of vitamin D [98].

Finally, vitamin D caused a reduction of oxidative stress induced by high glucose levels in a human renal tubular cell line through the upregulation of the AKT/UCP2 signaling pathway. These antioxidant effects were achieved by increasing the activity of superoxide dismutase and MMP and decreasing malondialdehyde levels [99].

In the same way, it has been shown that hypovitaminosis D may cause a decrease in the intracellular concentrations of glutathione, mediated by the enzyme c-glutamyl-transpeptidase, and a limited ability to reduce the levels of produced ROS. This pro-oxidative environment could favor the development of serious diseases such as multiple sclerosis [100]. In this sense, it has been suggested that during aging, people have a natural reduction in vitamin D serum levels, promoting a pro-oxidative state associated with mitochondrial dysfunction [101]. Lack of vitamin D also stimulates significant nitrosative stress at the brain level, which could promote cognitive impairment in middle-aged and older adults [102]. Therefore, those who have vitamin D deficiency are usually more susceptible to suffering from different pathologies associated with age such as Parkinson’s disease, Alzheimer’s, and cardiovascular disease, among others. On the other hand, subjects with normal plasma levels of vitamin D are generally less likely to suffer any of the diseases mentioned [103,104,105]. It has also been indicated that vitamin D deficiency is related to a decrease in the expression of VDR and an altered activity of antioxidant enzymes at the skeletal muscle level. Additionally, vitamin D modulates oxygen consumption at the mitochondrial level. Therefore, vitamin D deficiency reduces the oxygen consumption rate and stimulates mitochondrial dysfunction. In this context, it was suggested that all these alterations lead to an increased probability of developing muscle atrophy and that vitamin D supplements could prevent or reverse them [106,107]. These findings were confirmed in another study on muscle cells with oxidative stress induced by tert-butyl hydroperoxide, where vitamin D also showed potent antioxidant effects that are considered beneficial for muscle tissue homeostasis [108].

Of particular interest, VDR has a vital role in the antioxidant actions of vitamin D and its analogs. In this regard, VDR has proven to be a cardioprotective and self-defensive receptor since it reduces the oxidative stress generated during ischemia-reperfusion injury in mouse hearts through a mechanism dependent on metallothionein. VDR activation by different agonists inhibited mitochondrial impairment through the reduction in caspase-9 activation and mitochondrial cytochrome c release. These cardioprotective effects were attenuated when VDR was silenced. Conversely, the over-expression of VDR provoked a decrease in myocardial infarct size and enhanced cardiac function through attenuating oxidative stress, among other protective mechanisms [109]. The silencing of VDR in a spontaneously transformed aneuploid immortal keratinocyte cell line from adult human skin provoked reliable increases in the mitochondrial membrane potential, causing sensitization of these cells to oxidative stress. Moreover, it was found that transcription of the subunits II and IV of cytochrome c oxidase were significantly increased after VDR silencing, suggesting an essential role of vitamin D in the maintenance of normal mitochondrial function and anti-oxidation at the cellular level by VDR activation [110]. In the same type of cultured keratinocytes, but without VDR silencing and under the condition of oxidative stress, the treatment with both calcitriol and its analogs 20(OH)D3, 21(OH)pD, and calcipotriol induced the expression of antioxidant enzymes such as superoxide dismutase and catalase as well as the maintenance of mitochondrial membrane potential [111]. It has also been observed that in different diseases (renal, hepatic, cardiovascular, dermal, etc.), the deletion of VDR or vitamin D insufficiency promotes the increase in oxidative stress. Conversely, both the activation of VDR by different agonists and high levels of vitamin D provoke significant antioxidant effects. Of particular interest, it has been demonstrated that vitamin D, through VDR activation, causes a considerable increase in the expression of the alpha-klotho protein, an important antioxidant, and a circulating antiaging factor. Moreover, other analogs of vitamin D such as paricalcitol and doxercalciferol have also demonstrated several antioxidant effects in different cells and tissues by stimulating VDR [112].

## 7. Interrelation among Oxidative Stress, RAAS, and SARS-CoV-2 Infection

Renin–angiotensin–aldosterone system (RAAS) is formed by two axes, a beneficial antioxidant axis composed of angiotensin-converting enzyme (ACE)-2 and Ang1-7/Mas receptor, and a deleterious pro-oxidant axis composed of ACE and the Ang II/AT1 receptor. The latter causes a considerable increase in oxidative stress in both tissue and plasma level [113]. It is known that many Ang II signaling pathways are mediated, at least in part, by ROS such as O_2_^•−^ and mitochondria are the main subcellular source of O_2_^•−^ induced by Ang II [114]. Despite O_2_^•−^ having a very short life, it may rapidly react with nitric oxide (NO^•−^) and form peroxynitrite (ONOO^−^), a potent oxidant able to induce cell apoptosis or necrosis [115]. Aldosterone induces both hypertrophy and fibrosis at the myocardial level through the promotion of oxidative stress and inflammation [116]. Chronic RAAS activation may alter mitochondrial function, and consequently may increase oxidative stress derived by mitochondria. In fact, it has been proven that exogenous Ang II raises mitochondrial oxidative stress at renal level in rats with heart failure, and increases renal mitochondrial dysfunction in aged mice [117]. RAAS is also involved in vascular complications associated with diabetes, at least in part, through the induction of oxidative stress. In this sense, it was confirmed that the blockade of AT1 receptors in human coronary artery endothelial cells reduces the endoplasmic reticulum stress and superoxide anion production induced by high glucose levels [118]. It has been shown that RAAS is over-activated in patients with kidney injury and that aldosterone induces chronic kidney disease mainly through endoplasmic reticulum stress and oxidative stress [119]. RAAS plays a key role in the pathophysiology of hypertension due, among other factors, to the fact that causes a significant stimulation of oxidative stress at the systemic level [120,121]. The activation of RAAS observed after myocardial infarction or heart failure leads to exacerbated production of ROS derived by increased activity of the NADPH oxidase enzyme. This augmented oxidative stress causes dysfunction and apoptosis of endothelial cells [122]. Oxidative stress dependent of RAAS over-activation has also been related to heart fibrosis. In this regard, it has been shown that strong generation of ROS induced by Ang II triggers activation of the pro-fibrotic TGFβ1-Smad2/3 signaling pathway and consequent synthesis of collagen in cardiac cells [123]. Oxidative stress induced by an over-stimulated RAAS also increased DNA damage both in vitro and in vivo models of hypertension. Ang II is the main factor responsible for the pro-oxidative state that leads to accumulation of mutations in different organs affected by hypertension such as kidneys. For this reason, hypertensive patients with over-activated RAAS and high concentrations of Ang II would have an increased risk of developing renal cancer, in accordance with the results revealed by different epidemiological studies [124].

Additionally, SARS-CoV-2 infection and its complications are associated, among other factors, with redox imbalance or oxidative stress [5,125]. The elevation of cytokine levels produced by SARS-CoV-2 infection also contributes to exacerbation of oxidative stress observed in this severe acute respiratory syndrome [126,127,128]. Oxidative stress induced by viral infections increases the DNA methylation deficiency, probably provoking a greater ACE2 hypomethylation. The hypomethylation may lead to an exacerbated expression of genes that encode ACE2. As ACE2 is the target receptor to which SARS-CoV-2 binds to enter the cells, it is possible that hypomethylation caused by oxidative stress enhances viremia. In this context, a vicious cycle of demethylation and augmented oxidative stress could increase susceptibility to SARS-CoV-2 infection [129]. Moreover, the antioxidant capacity is usually lost with age, and this pro-oxidative state associated with aging could be one of the reasons for which elderly people are more susceptible to being affected by SARS-CoV-2 infection [130,131,132].

Of special interest, it is known that the main risk factors for morbidity and mortality by COVID-19 are pathologies where there exist an over-activated RAAS such as hypertension, diabetes, cancer, and obesity, among others. These evidences would reinforce the postulated relationship among oxidative stress, RAAS, and SARS-CoV-2 infection [8]. Therefore, synergistic oxidative stress resulting from an over-activated RAAS and the SARS-CoV-2 infection may contribute to create the “perfect storm”, which leads to known lethal consequences of this respiratory syndrome.

## 8. Vitamin D Antioxidative Actions against SARS-CoV-2 Infection

An inverse relationship has been established between vitamin D concentrations and the exacerbated oxidative stress associated with the RAAS activation, since lowered levels of vitamin D favor the over-activation of RAAS and vice versa [101,133,134,135]. Such over-activation is usually associated with elevated levels of renin, increased synthesis of Ang II [136,137], and augmented expression of ACE [138]. In this sense, it is known that both RAAS and VDR receptors are present at the mitochondrial level, mediating antagonistic effects [101,133,134,135]. VDR regulates both the nuclear (COX4 and ATP5B) and mitochondrial (COX2 and MT-ATP6) transcription of the proteins involved in ATP synthesis and respiratory activity. The activation of VDR localized in the mitochondrial compartment is responsible for cell metabolic control by reducing mitochondrial respiration and activating mitochondrial homeostatic processes. Thus, the low stimulation of VDR at the mitochondrial level in people with vitamin D deficiency may provoke mitochondrial dysfunction, an increased oxidative stress and, consequently, cell death [139,140]. In relation to mitochondrial RAAS, Abadir et al. showed the expression of Ang II type 2 receptors on mitochondrial inner membranes, which are colocalized with Ang II. Of interest, the aging process causes an increase in the expression of Ang II type 1 receptors at the mitochondrial level. Mitochondrial RAAS activation is associated with nitric oxide synthesis and an increase in oxidative stress [141]. The interplay between vitamin D and RAAS could explain, at least in part, the high incidence of cardiovascular pathologies in people with vitamin D deficiency [142]. Moreover, the normalization of vitamin D levels in patients with deficiency of this vitamin caused a blockade of peripheral RAAS [143]. In this regard, calcitriol has also been proven to be a modulator of liver RAAS, which is usually upregulated during insulin resistance [144]. It has also been found that activation of VDR causes the attenuation of acute lung injury by the RAAS blockade [145]. Long-term vitamin D deficiency may induce pulmonary fibrosis by exacerbated deposition of extracellular matrix at lung level due to an uncontrolled and chronic RAAS over-activation [146]. RAAS inhibition by vitamin D was also able to reduce vascular oxidative stress, exerting an important regulatory function on blood pressure modulation [147]. Paricalcitol has been demonstrated to decrease the expression of renin and Ang II induced by lipopolysaccharides at the hypothalamic level [148]. Vitamin D could also attenuate the complications associated with incident atrial fibrillation by RAAS inhibition [149] and normalize the exalted brain RAAS of 1α(OH)ase knockout mice by an antioxidant mechanism [150]. Vitamin D is able to suppress renin transcription mediated by VDR [151] and the activity blockade of Cyclic AMP [152] independently of calcium or phosphorus extracellular levels [153]. Klotho induction by vitamin D could also be a modulator mechanism of RAAS in order to avoid oxidative stress [154,155]. Exalted expression of Klotho reduces the production of many RAAS components including AT1, angiotensinogen, renin and ACE, among others [156]. Hence, vitamin D may significantly ameliorate the respiratory syndrome caused by SARS-CoV-2 infection not only through its own antioxidant effects, but also through RAAS inhibition. Additionally, in relation to vitamin D and RAAS facing to viral infections, it was observed that podocytes infected with HIV showed both downregulation of VDR and over-activation of RAAS with an enhanced renin expression and increased Ang II production. Vitamin D treatment reversed the RAAS over-activation and consequently free radical synthesis [157]. Similar results were obtained in tubular cells infected with HIV, where vitamin D confirmed its protective properties on cell damage induced by HIV [158]. In this sense, the results observed with HIV could be analogs to those obtained with SARS-CoV-2.

**Figure 2:**
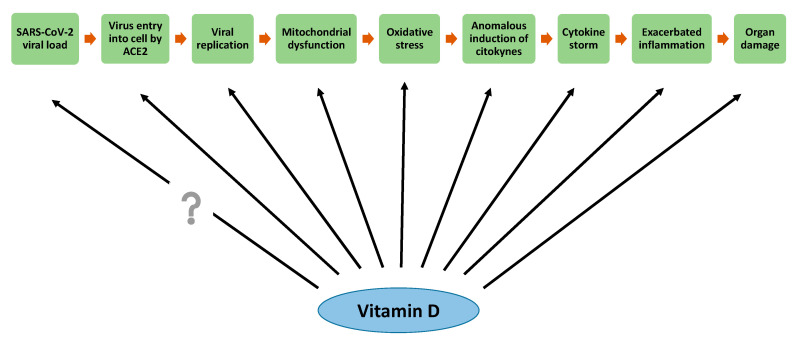
Possible participation of vitamin D on stages of SARS-CoV-2 infection.

SARS-CoV-2 infection may produce multiple free radicals such as H_2_O_2_, O_2_^•−^ and •OH, among the most prominent. Interactions between SARS-CoV-2 proteins and host cell mitochondrial proteins cause the loss of membrane integrity and mitochondrial dysfunction, which provokes an exacerbated increase in ROS production [159]. Although a certain level of ROS is important in the regulation of immune response and the elimination of virus, its exacerbated production may destroy many cell components in both virus-infected cells and normal cells, causing multiple organ failure [160]. As previously mentioned, vitamin D is able to increase the expression of antioxidant enzymes such as glutathione reductase. High levels of glutathione have a similar effect to vitamin C supplementation, which act as an antioxidant and antimicrobial. Therefore, it has been proposed that vitamin D could be useful in the prevention and treatment of COVID-19 [161]. As an antecedent at the respiratory level, vitamin D stimulated the activation of the glucose-6-phosphate dehydrogenase signaling pathway and the production of oxidized glutathione as antioxidant mechanisms against oxidative stress induced by pollutant particulate matter in human bronchial epithelial cells. Therefore, vitamin D may exert a protective antioxidant effect on lungs and airways under oxidative stress conditions such as asthma or chronic obstructive pulmonary disease in smoking patients [162,163]. In this regard, it was observed that patients with pulmonary pathologies usually have lowered vitamin D serum levels, which would indicate a possible correlation between an impaired antioxidant defense and a susceptibility to suffer lung disease [164]. Therefore, the antioxidant potential of vitamin D could attenuate COVID-19 complications, especially at pulmonary level (Figure 2).

There are no accurate data yet on the exact doses of vitamin D recommended to protect against COVID-19. Some authors recommend high doses for a short time or low doses for a longer time. For instance, Grant et al. recommended that people at risk of COVID-19 consider taking 10,000 IU/day of vitamin D3 for a month to rapidly increase their 25(OH)D concentrations, followed by 5000 IU/day during a few more weeks in order to reduce the infection risk [161]. On the other hand, other authors have suggested that single oral doses >500,000 IU of cholecalciferol are able to rapidly increase serum vitamin D concentrations in patients with severe vitamin D deficiency, without producing side effects [165]. Anyway, the aim should be to raise vitamin D concentrations above 40–60 ng/mL, which would be the range of protection by vitamin D against respiratory infections [161].

## 9. Conclusions and Prospect

In general, viruses including SARS-CoV-2 usually alter mitochondrial dynamics at different levels for achieving the progression of infection. Some of these mechanisms include mitochondrial DNA damage, changes in mitochondrial membrane potential, alterations in mitochondrial metabolic pathways and calcium homeostasis, modifications in number and distribution of mitochondria into the cells, impairment of the body’s antioxidant defense, and increases in ROS levels, among others.

**Figure 3:**
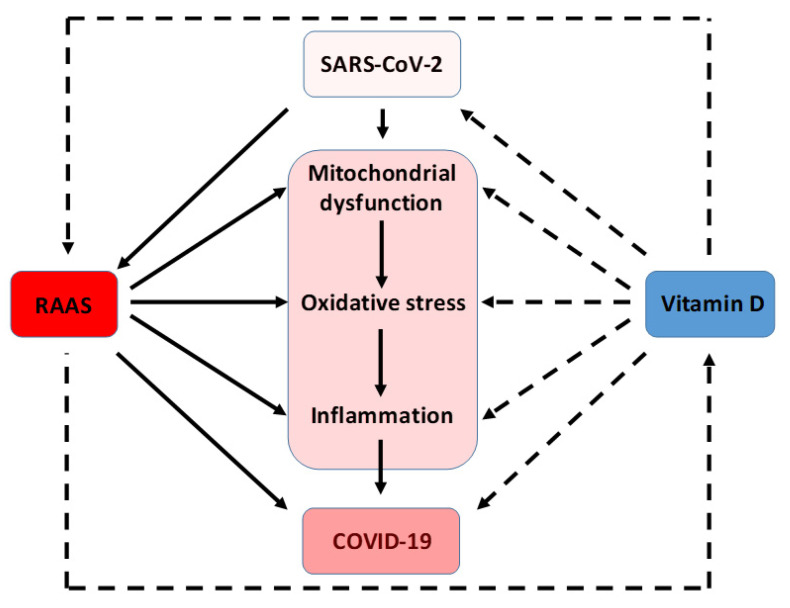
Interplay between mitochondrial dysfunction, RAAS over-activation, and vitamin D levels in the physiopathology of COVID-19. Solid lines indicate stimulation/induction, while dashed lines indicate inhibition/blocking.

On the other hand, it is known that vitamin D is a hormone that may act as a potent anti-inflammatory and antioxidant. It has also been observed that some of the individuals most susceptible to being infected by SARS-CoV-2 are those who have some underlying pathology associated with the over-activation of RAAS (obese, aged, male, hypertension, diabetic, and others). This activation causes an increased production of ROS. Vitamin D is able to reduce the RAAS activation and consequently decrease ROS generation and to improve the prognosis of SARS-CoV-2 infection (Figure 2 and Figure 3).

As a future prospect, we propose improving the vitamin D plasma levels of the world population, especially of those individuals with additional risk factors that predispose them to the lethal consequences of SARS-CoV-2 infection.
